# Assessing the diagnostic value of a potential screening tool for detecting early interstitial lung disease at the onset of inflammatory rheumatic diseases

**DOI:** 10.1186/s13075-022-02786-x

**Published:** 2022-05-12

**Authors:** Tobias Hoffmann, Peter Oelzner, Marcus Franz, Ulf Teichgräber, Diane Renz, Martin Förster, Joachim Böttcher, Claus Kroegel, P. Christian Schulze, Gunter Wolf, Alexander Pfeil

**Affiliations:** 1 Department of Internal Medicine III, Jena University Hospital – Friedrich Schiller University Jena, Am Klinikum 1, 07747 Jena, Germany; 2 Department of Internal Medicine I, Jena University Hospital – Friedrich Schiller University Jena, Am Klinikum 1, 07747 Jena, Germany; 3 Institute of Diagnostic and Interventional Radiology, Jena University Hospital – Friedrich Schiller University Jena, Am Klinikum 1, 07747 Jena, Germany; 4 Institute of Diagnostic and Interventional Radiology, Department of Pediatric Radiology, Hannover Medical School, Carl-Neuberg-Str. 1, 30625 Hannover, Germany

**Keywords:** Inflammatory rheumatic disease, IRD, Interstitial lung disease, ILD, Screening, Pulmonary function test, PFT, Chest X-ray, High-resolution computed tomography, HRCT

## Abstract

**Background:**

Interstitial lung disease (ILD) is a severe pulmonary complication in inflammatory rheumatic diseases (IRD) and associated with significantly increased morbidity and mortality. That is why ILD screening at a very early stage, at the onset of IRD, is essential. The objective of the present study was to evaluate the diagnostic value and utility of a stepwise approach as a potential ILD screening tool in patients with newly diagnosed IRD.

**Methods:**

Consecutively, 167 IRD patients were enrolled. To homogenize the study cohort, an age and gender matching was performed. The case-control study included 126 patients with new onset of IRD (mainly connective tissue diseases [CTD], small vessel vasculitis, and myositis). We applied a stepwise screening algorithm in which all patients underwent pulmonary function testing (PFT) and/or additional chest radiography. If there was at least one abnormal finding, pulmonary high-resolution computed tomography (HRCT) was subsequently performed.

**Results:**

With our stepwise diagnostic approach, we identified 63 IRD patients with ILD (ILD group) and 63 IRD patients without ILD (non-ILD group). A reduced diffusing capacity for carbon monoxide (DLCO) < 80% showed a sensitivity of 83.6% and a specificity of 45.8% compared to chest X-ray with 64.2% and 73.6%, respectively, in detecting ILD. The combination of reduced DLCO and chest X-ray revealed a sensitivity of 95.2% and a specificity of 38.7%. The highest sensitivity (95.2%) and specificity (77.4%) were observed for the combination of reduced DLCO, chest X-ray, and pulmonary HRCT. The most common pulmonary abnormalities on HRCT were ground-glass opacities (GGO; 36.5%), followed by non-specific interstitial pneumonia (NSIP; 31.8%) and usual interstitial pneumonia (UIP; 9.5%).

**Conclusions:**

The combination of reduced DLCO (< 80%), chest X-ray, and pulmonary HRCT yielded the highest sensitivity and specificity in detecting ILD at the onset of IRD. Therefore, this stepwise approach could be a new screening algorithm to identify IRD patients with pulmonary involvement already at the time of the initial IRD diagnosis.

## Introduction

Based on growing insights into the immunopathological pathways, rheumatology has changed over the years from a discipline that focused mainly on joint diseases to a wide spectrum of inflammatory rheumatic diseases (IRD), encompassing inflammatory joint diseases, connective tissue diseases (CTD), and myositis as well as vasculitis [1–6].

Many IRD present with complex clinical pictures, involving other tissues, of which the lungs are a frequent target of autoimmune-mediated injury (10–65% depending on the disease) [7–12]. Among many diverse types of IRD-associated lung involvements, the most common is interstitial lung disease (ILD) which clinical manifestations and severity can vary from subclinical abnormality to dyspnea, respiratory failure, and death [13–15]. ILD in IRD is associated with a significant morbidity [16, 17] and, for example, the leading cause of mortality in SSc patients, accounting for approximately 35% of SSc-related deaths [18] and a mortality risk nearly three times greater than SSc patients without ILD [19].

Therefore, effective screening to improve the early diagnosis of IRD patients with associated ILD is of paramount importance [13, 14]. Most published data relate to the detection of ILD in the presence of longer-established IRD (e.g., within the first 3–5 years after SSc diagnosis) with minimal standardized screening recommendations for ILD in patients with CTD [20]. Given the poor prognosis and the availability of a new therapeutic option (nintedanib) in pulmonary fibrosis in rheumatic systemic diseases, an organ screening should be performed much earlier—at the time of the initial diagnosis of an IRD—to detect pulmonary involvement. As shown in various studies, 54 to 65% of patients with SSc or dermatomyositis presented with lung involvement at the onset of their disease [8, 21]. However, only less than half of SSc patients underwent a basic organ screening at the time of initial diagnosis, as shown in a survey with members of the Scleroderma Society of Canada [22].

Therefore, the aim of our study was to determine the value of a stepwise diagnostic screening approach for ILD in IRD. All patients with an initial diagnosis of IRD underwent pulmonary function testing (PFT) and chest radiography. In case of at least one pathologic finding, a pulmonary high-resolution computed tomography (HRCT) was subsequently performed.

## Patients and methods

### Patients

Consecutively, 167 patients (127 women and 40 men, mean age 54.7 ± 15.3 years) were maintained at the onset of IRD between 2005 and 2020. All participants were examined and treated at the Department of Rheumatology, University Hospital Jena/Germany. Based on the diagnostic procedure, 68 patients with ILD and 99 patients without ILD were identified.

Based on the heterogeneity concerning age and sex of the study cohort, an age and gender matching was performed to homogenize and standardized the study cohort using the concept of a case-control study. The case-control study encompasses 126 patients (ILD group *n* = 63, non-ILD group *n* = 63).

All patients were diagnosed with newly IRD on the basis of a comprehensive rheumatologic assessment; no patient has been previously evaluated for ILD. The exclusion criteria were defined as (I) already known diagnosed IRD, (II) immunosuppressive treatment, or (III) antifibrotic treatment. There were no exclusion criteria for other pulmonary pre-existing diseases. The diagnosis of ILD in IRD was performed in a consensus panel by rheumatologists, pulmonologists, and radiologists using the clinical, laboratory, and imaging findings.

IRD encompassed connective tissue disease ([CTD], systemic lupus erythematosus [SLE], SSc, *Sjögren’s syndrome*, Sharp syndrome), small vessel vasculitis (granulomatosis with polyangiitis [GPA], microscopic polyangiitis [MPA], eosinophilic granulomatosis with polyangiitis [EGPA]) and myositis (dermatomyositis, polymyositis, necrotizing myositis, Jo1-anti-synthetase syndrome).

### Methods

#### Medical history and clinical examination

A detailed medical history was taken in all patients with regard to clinical ILD symptoms including cough, dyspnea, and sputum. On physical examination, lung auscultation focussed on inspiratory crepitations (sclerosiphonia). In addition, pulmonary comorbidities (chronic obstructive pulmonary disease [COPD] and severe emphysema) and smoking status were documented.

#### PFT and chest X-ray

All patients underwent PFT with forced expiratory volume in 1 s (FEV_1_), forced vital capacity (FVC), total lung capacity (TLC), transfer factor of the lung for carbon monoxide (TLCO), and diffusing capacity for carbon monoxide (DLCO). DCLO < 80% was considered as a reduced diffusing capacity. In addition, a chest X-ray was also performed.

#### HRCT

Patients with at least one suspicious finding in PFT (in case of DLCO < 80%) or chest X-ray (findings reported as suspicious for ILD by the radiologist) underwent pulmonary HRCT, using the standard protocol of the manufacturers. All scans were analyzed with respect to parenchymal changes (including ground-glass opacities [GGO] and granuloma/proliferations) in lung window images and evaluated in collaboration with two chest radiologists and a rheumatologist according to the recommendations/criteria by the American Thoracic Society/European Respiratory Society and the Fleischner Society White Paper [23–25].

Additionally, each chest X-ray and HR-CT were scored by a radiologist and a rheumatologist in consensus. In the case of ambiguity, a second radiologist reviewed the radiographs or HRCT. The radiologists were experts in ILD and each has experience > 15 years.

### Statistical analysis

The data were documented in Microsoft Excel® (Microsoft Windows, Redmond, WA, USA). The statistical analysis was performed by IBM SPSS Statistics 25 (IBM SPSS Statistics, Chicago, IL, USA, for Windows). The statistical analysis included the following steps:

I. At the beginning, a case-control matching was performed with the support of IBM SPSS Statistics 25. It was matched by gender and age. The tolerance/fuzz factor for age was 40.
II. In the following, a descriptive statistic was used to evaluate the data.
III. The sensitivity and specificity were verified by crosstabs and receiver operating characteristic (ROC) curve analysis. Considering that it is a case-control study, the prevalence independent positive and negative likelihood ratio (LR) was used. The area under the curve (AUC) was used to summarize the diagnostic accuracy of the evaluated diagnostic test. According to Hosmer, a value of 0.5 suggest no discrimination by the test, 0.7 to 0.8 is acceptable, 0.8 to 0.9 is excellent, and > 0,9 is considered outstanding [26].
IV. Moreover, the following statistical tests were used to verify the differences and to objectify correlations: *t*-test (*t*) and Pearson’s chi-squared test (*χ*^2^) for independent samples. As correlation coefficients: Cramer’s *V* as a measure of the strength of the relationship between more than two dichotomous characteristics. According to Cohen, for the correlation coefficient (Cramer’s *V*): small effect size 0.1 to < 0.3, medium effect size 0.3 to < 0.5, and large effect size ≥ 0.5 [27]. A *P* < 0.05 was considered as statistically significant.
V. To verify the results of the case-control study on a representative clinical collective, the sensitivity, specificity, positive predictive value, and negative predictive value were evaluated using ROC curve analysis on 167 consecutive IRD patients.

## Results

### Baseline characteristics (see Tables 1 and 2)

The study included 126 patients with the initial diagnosis of IRD. With our stepwise diagnostic approach, we identified 63 IRD patients with ILD and 63 IRD patients without ILD (non-ILD group). Due to case-control matching, there were no significant differences in age, gender, or the performance of PFT or chest X-ray. However, in the ILD group, there were significant more participants with small vessel vasculitis (ILD group: *N* = 16 [25.4%]; non-ILD group: *N* = 3 [4.8%]) and myositis (ILD group: *N* = 12 [19.0%]; non-ILD group: *N* = 5 [7.9%]). Cramer’s *V* shows a medium effect size.

**Table 1:** Baseline characteristics of the ILD group and non-ILD group

Variable	ILD group, number (%)	Non-ILD group, number (%)	Difference
**Number**	63 (50.0%)	63 (50.0%)	
**Gender**			
Men	17 (27.0%)	17 (27.0%)	*χ*^*2*^*(1) = 0.000,****P = 1.000***
Women	46 (63.0%)	46 (63.0%)	
**Age**			
Median ± SD	58.6 ± 14.2 years	53.8 ± 14.3 years	*t(124) = − 1.907,****P = 0.059***
**Inflammatory rheumatic diseases**			
Connective tissue disease	35 (55.6%)	55 (87.3%)	*χ*^*2*^*(3) = 17.732,****P < 0.001****, Cramer’s V = 0.375*
Small vessel vasculitis	16 (25.4%)	3 (4.8%)	
Myositis	12 (19.0%)	5 (7.9%)	
**Symptoms**			
Dyspnea	36 (57.1%)	22 (34.9%)	*χ*^*2*^*(1) = 6.262,****P = 0.012***
Cough	17 (27.0%)	11 (17.5%)	*χ*^*2*^*(1) = 1.653,****P = 0.199***
Sputum	11 (17.5%)	8 (12.7%)	*χ*^*2*^*(1) = 0.558,****P = 0.455***
Sclerosiphonia	20 (31.7%)	5 (7.9%)	*χ*^*2*^*(1) = 11.228,****P = 0.001***
No symptoms	20 (31.7%)	30 (47.6%)	*χ*^*2*^*(1) = 3.316,****P = 0.069***
**Pulmonary comorbidities**			
Emphysema	2 (3.2%)	3 (4.8%)	*χ*^*2*^*(1) = 0.19,****P = 0.661***
COPD	3 (4.8%)	1 (1.6%)	*χ*^*2*^*(1) = 1.03,****P = 0.310***
Smoking			
Active	10 (15.9%)	11 (17.5%)	*χ*^*2*^*(3) = 4.92,****P = 0.085***
Ex-smoker	18 (28.6%)	8 (12.7%)	
Pack years	21.7 ± 12.3	22.7 ± 13.9	*t(38) = − 0.24,****P = 0.808***

**Table 2:** Distribution of IRD in the ILD group and non-ILD group

Inflammatory rheumatic diseases	ILD group	Non-ILD group
**Connective tissue disease**	**35 (55.6%)**	**55 (87.3%)**
Systemic lupus erythematosus	4	15
Systemic sclerosis	16	19
Sjögren’s syndrome	9	17
Sharp syndrome	6	4
**Small vessel vasculitis**	**16 (25.4%)**	**3 (4.8%)**
Granulomatosis with polyangiitis	7	1
Microscopic polyangiitis	3	1
Eosinophilic granulomatosis with polyangiitis	6	1
**Myositis**	**12 (19.0%)**	**5 (7.9%)**
Myositis/polymyositis	0	1
Dermatomyositis	4	2
Jo1-anti-synthetase syndrome	8	1
Necrotizing myositis	0	1
**Total**	**63 (100.0%)**	**63 (100.0%)**

In total, 60.3% (*N* = 76) of patients showed pulmonary symptoms, but there was no significant difference regarding symptomatic and asymptomatic patients (*P* = 0.069) (see Table 1). Furthermore, ILD patients presented with a significant higher rate of dyspnea (57.1%) and sclerosiphonia (31.7%), compared to non-ILD patients (dyspnea 34.9%, sclerosiphonia 7.9%, *P* < 0.05).

Regarding pulmonary comorbidities, there was no significant difference in COPD (ILD group *n* = 3, non-ILD group *n* = 1, p = n. s.) and emphysema (ILD group *n* = 2, Non-ILD group *n* = 3, *P* = n. s.). There was also no significant difference for smoking status (active smoker: ILD group *n* = 10, non-ILD group *n* = 11, *P* = n. s.; ex-smoker: ILD group *n* = 18, non-ILD group *n* = 8, *P* = n. s.) or pack years (ILD group 21.7 ± 12.3, non-ILD group 22.7 ± 13.9, *P* = n. s.).

### PFT (see Tables 3 and 4)

For DLCO < 80%, the sensitivity and specificity for the detection of ILD in patients with IRD were 83.6% and 45.8%, respectively. DLCO < 70% revealed a sensitivity and specificity of 67.2% and 76.3%, respectively. Regarding FVC < 80%, FEV_1_ < 80%, TLC < 80%, and TLCO < 80%, a lower sensitivity with a higher specificity was observed. The highest area under the curve (AUC) was achieved by DLCO (0.772). Moreover, the lowest negative LR was observed by DLCO < 80% (0.36).

**Table 3:** Pulmonary function tests, chest X-ray, HRCT, and origin of pathologies in HRCT, differentiated regarding the ILD group and non-ILD group

Variable	ILD group, number (%)	Non-ILD group, number (%)	Difference
**Number**	63 (50.0%)	63 (50.0%)	
**PFT**	61 (96.8%)	61 (96.8%)	*χ*^*2*^*(1) = 0.000,****P = 1.000***
Median ± SD			
DLCO	59.4 ± 19.6%	79.9 ± 18.3%	*t(118) = 5.91,****P < 0.001***
TLC	84.4 ± 19.8%	98.5 ± 12.9%	*t(95) = 4.47,****P < 0.001***
FVC	82.3 ± 21.3%	92.6 ± 17.9%	*t(118) = 2.89,****P = 0.005***
FEV_1_	82.0 ± 23.4%	92.2 ± 16.6%	*t(104) = 2.78,****P = 0.007***
TLCO	75.0 ± 19.6%	88.1 ± 17.3%	*t(116) = 3.84,****P < 0.001***
**Chest X-ray**	53 (84.1%)	53 (84.1%)	*χ*^*2*^*(1) = 0.000,****P = 1.000***
**HRCT**	63 (100.0%)	38 (60.3%)	*χ*^*2*^*(1) = 43.955,****P < 0.001***
**Origin of the pathologies in HRCT**			
ILD in IRD	63 (100.0%)	0 (0.0%)	*χ*^*2*^*(4) = 80.000,****P < 0.001***
Respiratory bronchiolitis ILD	0 (0.0%)	7 (11.1%)	
Post-inflammatory change	0 (0.0%)	6 (9.5%)	
Other lung diseases	0 (0.0%)	2 (3.2%)	

*DLCO* Diffusing capacity for carbon monoxide, *FEV*_*1*_ Forced expiratory volume in 1 s, *FVC* Forced vital capacity, *HRCT* High-resolution computed tomography, *LR* Likelihood ratio, *PFT* Pulmonary function tests, *TLC* Total lung capacity, *TLCO* Transfer factor of the lung for carbon monoxide

**Table 4:** Area under the curves (AUC), sensitivity, specificity, positive likelihood ratio (LR+), and negative likelihood ratio (LR−) for different cutoffs in the detection of lung involvement in IRD patients by different examinations in the ILD group

Diagnostic procedure	Parameter	AUC (95% CI; ***P***)	Cutoff	Sensitivity	Specificity	LR+	LR−
**PFT**	DLCO	0.772 (0.690–0.855; *P* < 0.001)	< 80%	83.6%	45.8%	1.54	0.36
			< 70%	67.2%	76.3%	2.84	0.43
	TLC	0.707 (0.610–0.803; *P* < 0.001)	< 80%	32.1%	94.6%	5.94	0.72
			< 70%	23.2%	100.0%	> 100	0.77
	TLCO	0.686 (0.591–0.781; *P* = 0.001)	< 80%	57.6%	67.8%	1.79	0.63
			< 70%	32.2%	84.7%	2.10	0.80
	FVC	0.648 (0.548–0.747; *P* = 0.005)	< 80%	47.5%	78.7%	2.23	0.67
			< 70%	32.2%	91.8%	3.93	0.74
	FEV_1_	0.629 (0.526–0.732; *P* = 0.015)	< 80%	49.2%	82.0%	2.73	0.62
			< 70%	33.9%	91.1%	3.81	0.73
**Chest X-ray**				64.2%	73.6%	2.43	0.49
**HRCT**				100.0%	55.3%	2.24	< 0.01

*DLCO* Diffusing capacity for carbon monoxide, *FEV*_*1*_ Forced expiratory volume in 1 s, *FVC* Forced vital capacity, *HRCT* High-resolution computed tomography, *LR* Likelihood ratio, *PFT* Pulmonary function tests, *TLC* Total lung capacity, *TLCO* Transfer factor of the lung for carbon monoxide

Regarding the differentiation of IRD subgroups, the highest sensitivity with 91.7% and specificity of 45.8% was evaluated for DLCO < 80% in patients with myositis. Participants with small vessel vasculitis showed the lowest sensitivity (66.7%) and specificity (45.8%).

### Chest X-ray (see Tables 3 and 4)

Chest X-ray revealed a sensitivity of 64.2% and a specificity of 73.6% in detecting ILD in IRD patients. For IRD subgroups, the sensitivity was 63.3% for CTD patients, 61.5% for small vessel vasculitis, and 70.0% for myositis with a specificity of 73.6% for all three aetiologies.

### Pulmonary HRCT findings at the onset of IRD (see Fig. 1)

The most common pulmonary abnormalities on HRCT were GGO (in total 28.6% of patients; ILD group: 36.5%; non-ILD group: 20.6%, *P* < 0.01), followed by non-specific interstitial pneumonia (NSIP) (in total 16.7%; ILD group: 31.8%; non-ILD group: 1.6%, *P* < 0.01), usual interstitial pneumonia (UIP) (in total 6.3%; ILD group: 9.5%; non-ILD group: 3.2%, *P* < 0.01), and probable UIP (in total 5.6%; ILD group: 11.1%; non-ILD group: 0%, *P* < 0.01). Finally, 33.3% of patients in the non-ILD group showed no lung parenchyma abnormality on HRCT. The sensitivity and specificity of HRCT in detecting IRD-ILD were 100.0% and 55.3%, respectively.

**Fig. 1:**
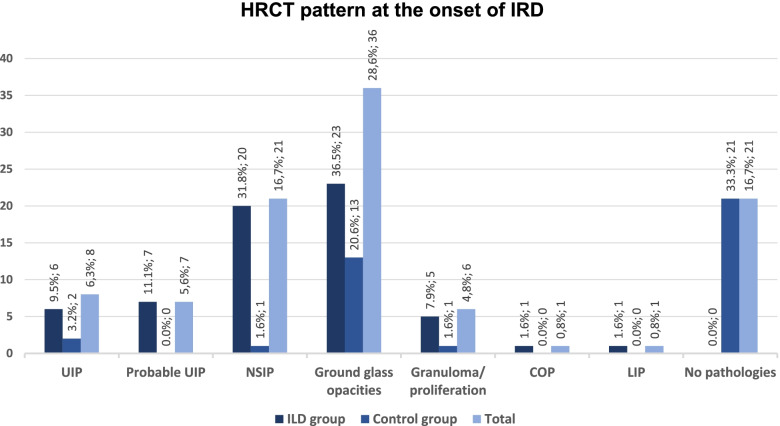
HRCT pattern at the onset of ILD in IRD patients. COP - cryptogenic organizing pneumonia; LIP - lymphoid interstitial pneumonia; NSIP - non-specific interstitial pneumonia; UIP - usual interstitial pneumonia

### Combination of DLCO and chest X-ray (see Table 5 and Fig. 2)

The combination of DLCO < 80% and chest X-ray resulted in a sensitivity and specificity of 95.2% and 38.7%. In patients with CTD, small vessel vasculitis, and myositis, chest X-ray combined with DLCO was associated with an increase of sensitivity (CTD 94.1%, small vessel vasculitis 93.8%, myositis 100.0%) with a specificity of 38.7%. By using this combination, a negative likelihood ratio of 0.12 with a positive likelihood ratio of 1.55 could be achieved (see Table 5). Consequently, a negative test result is 8.3 times more likely in non-ILD patients than in ILD patients.

**Table 5:** Sensitivity, specificity, positive likelihood ratio (LR+), and negative likelihood ratio (LR−) for different combinations and cutoffs of diagnostic procedures in the detection of lung involvement in IRD patients with ILD

Diagnostic procedure	Parameter	Cutoff	Sensitivity	Specificity	LR+	LR−
**Combination** of **PFT** and **chest X-ray**	DLCO and/or chest X-ray	< 80%	95.2%	38.7%	1.55	0.12
		< 70%	88.7%	64.5%	2.50	0.18
	TLC and/or chest X-ray	< 80%	69.4%	73.8%	2.65	0.41
		< 70%	64.5%	77.0%	2.80	0.46
	TLCO and/or chest X-ray	< 80%	78.7%	58.1%	1.88	0.37
		< 70%	68.9%	69.4%	2.25	0.45
	FVC and/or chest X-ray	< 80%	80.6%	66.1%	2.38	0.29
		< 70%	71.0%	74.2%	2.75	0.39
	FEV_1_ and/or chest X-ray	< 80%	80.6%	66.1%	2.38	0.29
		< 70%	71.0%	74.2%	2.75	0.39
**Combination** of **PFT** and **HRCT**	DLCO and HRCT	< 80%	83.6%	83.1%	4.95	0.20
		< 70%	67.2%	88.1%	5.65	0.37
	TLC and HRCT	< 80%	32.1%	98.2%	17.83	0.69
		< 70%	23.2%	100.0%	> 100	0.77
	TLCO and HRCT	< 80%	57.6%	81.4%	3.10	0.52
		< 70%	32.2%	91.5%	3.79	0.74
	FVC and HRCT	< 80%	47.5%	93.4%	7.20	0.56
		< 70%	32.2%	95.1%	6.57	0.71
	FEV_1_ and HRCT	< 80%	50.8%	93.4%	7.70	0.53
		< 70%	33.9%	95.1%	6.92	0.70
**Combination** of the following: **1. PFT** and **chest X-ray** **2. HRCT**	1. DLCO and/or chest X-ray 2. HRCT	< 80%	95.2%	77.4%	4.21	0.06

*DLCO* Diffusing capacity for carbon monoxide, *FEV*_*1*_ Forced expiratory volume in 1 s, *FVC* Forced vital capacity, *HRCT* High-resolution computed tomography, *LR* Likelihood ratio, *PFT* Pulmonary function tests, *TLC* Total lung capacity, *TLCO* Transfer factor of the lung for carbon monoxide

**Fig. 2:**
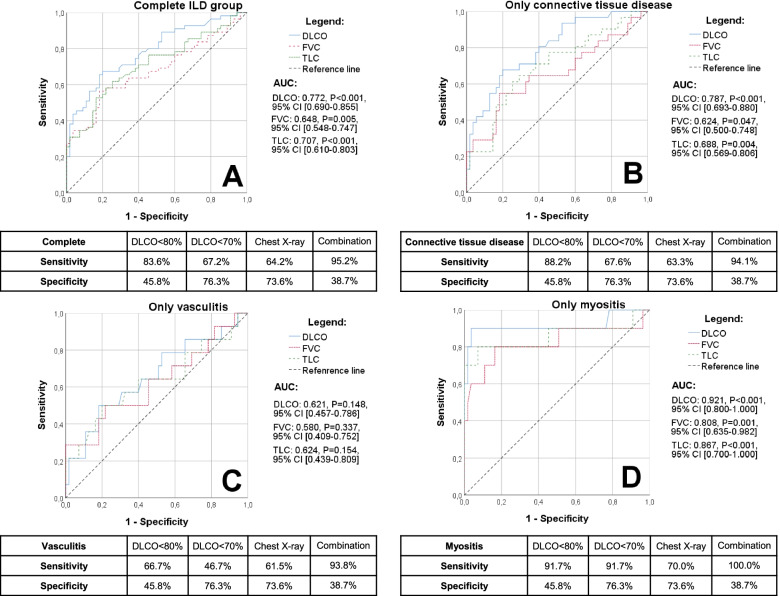
ROC curve analysis as well as sensitivity and specificity of different cutoffs in DLCO, chest X-ray, and a combination (DLCO < 80% or/and pathological findings in chest X-ray) in relation to subpopulations of the ILD group. **A** Complete ILD group. **B** CTD group. **C** Vasculitis group. **D** Myositis group

### Combination of DLCO, chest X-ray, and pulmonary HRCT (see Table 5 and Fig. 2)

If the DLCO (< 80%) and the first chest X-ray were combined and added to the following pulmonary HRCT (if ≥ 1 pathologic finding present), a sensitivity and a specificity of 95.2% and 77.4%, respectively, were achieved. This stepwise approach obtains a negative likelihood ratio of 0.06 and a positive likelihood ratio of 4.12.

### Sub-analysis with pulmonary HRCT in all patients (see Table 6)

Because pulmonary HRCT is the gold standard for diagnosing ILD in IRD, a sub-analysis was performed, in which each patient received pulmonary HRCT (see Table 6). The sub-analysis encompassed 76 patients (38 IRD patients with ILD and 38 IRD patients without ILD). There were no significant differences in the baseline characteristics. The DLCO, FVC, FEV_1_, and TLC revealed the significant differences in group comparisons. For DLCO < 80%, the sensitivity and specificity for the detection of ILD in IRD were 86.5% and 36.1%, respectively. Regarding FVC < 80%, FEV_1_ < 80%, TLC < 80%, and TLCO < 80%, a lower sensitivity with a higher specificity was observed. The highest area under the curve (AUC) was also achieved by DLCO (0.745).

**Table 6:** Sub-analysis of patients who all received a pulmonary HRCT. Baseline characteristics and sensitivity, specificity, positive likelihood ratio (LR+), negative likelihood ratio (LR−), and area under the curve (AUC) for different diagnostic procedures in the detection of lung involvement in IRD patients with ILD

Variable	ILD group, number (%)	Non-ILD group, number (%)	Difference	AUC
**Number**	38 (50.0%)	38 (50.0%)		
**Age**	59.7 ± 15.0 years	53.2 ± 14.7 years	*t(74) = 1.91,****P = 0.060***	
**PFT**	37 (97.4%)	37 (97.4%)	*χ*^*2*^*(1) = 0.000,****P = 1.000***	
Median ± SD				
DLCO	57.8 ± 19.3%	76.0 ± 19.7%	*t(71) = 3.99,****P < 0.001***	0.745 (0.633–0.857; *P* < 0.001)
	DLCO < 80%: sensitivity = 86.5%, specificity = 36.1%, LR+ = 1.35, LR− = 0.37			
TLC	82.7 ± 20.8%	97.4 ± 12.9%	*t(51.1) = 3.49,****P = 0.001***	0.724 (0.598–0.849; *P* = 0.002)
	TLC < 80%: sensitivity = 39.4%, specificity = 94.1%, LR+ = 6.70, LR− = 0.64			
FVC	78.9 ± 22.2%	91.3 ± 16.4%	*t(64.5) = 2.70,****P = 0.009***	0.667 (0.539–0.794; *P* = 0.014)
	FVC < 80%: sensitivity = 55.6%, specificity = 78.5%, LR+ = 2.57, LR− = 0.57			
FEV_1_	80.5 ± 23.5%	91.1 ± 14.3%	*t(55.5) = 2.30,****P = 0.025***	0.649 (0.517–0.782; *P* = 0.029)
	FEV_1_ < 80%: sensitivity = 54.3%, specificity = 78.4%, LR+ = 2.51, LR− = 0.58			
TLCO	74.6 ± 18.5%	82.8 ± 17.4%	*t(97) = 1.93,****P = 0.058***	0.615 (0.485–0.746; *P* = 0.095)
	TLCO < 80%: sensitivity = 60.0%, specificity = 55.6%, LR+ = 1.35, LR− = 0.72			
**Chest X-ray**	29 (76.3%)	29 (76.3%)	*χ*^*2*^*(1) = 0.000,****P = 1.000***	
	Sensitivity = 72.4%, specificity = 58.6%, LR+ = 1.75, LR− = 0.47			
**HRCT**	38 (100.0%)	38 (100.0%)	*χ*^*2*^*(1) = 0.000,****P = 1.000***	
	Sensitivity = 100.0%, specificity = 52.6%, LR+ = 2.11, LR− = < 0.01			
**Combination of chest X-ray and/or**				
DLCO < 80%	Sensitivity = 89.5%, specificity = 26.3%, LR+ = 1.21, LR− = 0.40			
DLCO < 70%	Sensitivity = 84.2%, specificity = 50.0%, LR+ = 1.68, LR− = 0.32			
FVC < 80%	Sensitivity = 78.9%, specificity = 57.9%, LR+ = 1.88, LR− = 0.36			
FVC < 70%	Sensitivity = 71.1%, specificity = 65.8%, LR+ = 2.08, LR− = 0.44			
**Combination** of the following: **1. DLCO < 80%** and/or **chest X-ray** and **2. HRCT**	Sensitivity = 89.5%, specificity = 65.8%, LR+ = 2.62, LR− = 0.16			

*DLCO* Diffusing capacity for carbon monoxide, *FEV*_*1*_ Forced expiratory volume in 1 s, *FVC* Forced vital capacity, *HRCT* High-resolution computed tomography, *LR* Likelihood ratio, *PFT* Pulmonary function tests, *TLC* Total lung capacity, *TLCO* Transfer factor of the lung for carbon monoxide

The combination of DLCO < 80% and chest X-ray resulted in a sensitivity and a specificity of 89.5% and 26.3%, respectively, with a negative LR of 0.40 could be achieved. With a modified cutoff (DLCO < 70%), the negative LR was reduced to 0.32 (sensitivity of 84.2%, specificity of 50.0%).

### Chest X-ray, PFT, and pulmonary HRCT on a representative clinical collective

The representative collective encompassed 167 patients (127 women and 40 men, mean age 54.7 ± 15.3), 68 patients with ILD and 99 patients without ILD. The AUC of PFT parameter are as follows: DLCO − 0.783; 95% CI 0.708–0.859; *P* ≤ 0.001 and FVC − 0.664; 95% CI 0.572–0.756; *P* = 0.001. This results in the following sensitivities and specificities for the selected cut-offs: DLCO < 80% — sensitivity 83.6%, specificity 48.3%, PPV 0.53, NPV 0.81; FVC < 80% — sensitivity 47.5%, specificity 79.8%, PPV 0.61, NPV 0.70.

The combination of DLCO < 80% and chest X-ray resulted in a sensitivity and a specificity of 94.1% and 47.5%, respectively, with a PPV of 0.55 and NPV of 0.92 could be achieved. In additional combination with the HRCT as a second step, a sensitivity and a specificity of 92.6% and 82.8%, respectively, could be achieved, with PPV of 0.79 and NPV of 0.94.

## Discussion

The aim of the present study was to determine the value of a stepwise diagnostic screening approach, using PFT, chest radiography, and pulmonary HRCT for detecting ILD in newly diagnosed patients with IRD.

Given the high mortality in IRD and pulmonary manifestations and the availability of the new therapeutic option nintedanib for other chronic fibrosing ILD with a progressive phenotype beyond idiopathic pulmonary fibrosis (IPF) and SSc-ILD, an early pulmonary screening at the time of IRD diagnosis is essential and meaningful.

### PFT

Caron et al. and Nihtyanova et al. showed that a reduced DLCO (< 80%) is associated with lung complications in patients with IRD [28, 29]. In our present study, DLCO < 80% revealed a sensitivity of 83.6% and a specificity of 45.8% for the detection of ILD in patients with IRD. This was in accordance with the data reported by Bernstein et al. yielding a sensitivity of 80.0% and specificity of 51.0% in detecting ILD in early SSc [21]. In addition, different studies showed similar sensitivities and specificities for other PFT parameters: Showalter et al. and Suliman et al. demonstrated a sensitivity and a specificity of 37.5 to 69.0% and 73.0 to 92.0%, respectively, for FVC < 80% [30, 31]. According to Newall et al., there were no significant differences for FVC, TLCO, or FEV_1_ between patients with or without ILD [32]. Rosenberg et al. showed sensitivities of 55% (FEV_1_) and 41% (FVC) [33]. Even the sub-analysis (only patients with pulmonary HRCT) showed no substantial changes in sensitivities and specificities. Consequently, FVC, TLCO, or FEV_1_ cannot be used to diagnose IRD-ILD. Additionally, our study showed that IRD patients without symptoms and a reduced DLCO (< 80%) presented IRD-ILD in 19.0% (*N* = 12) of cases and 23.8% (*N* = 15) had no ILD.

### Chest X-ray

We showed that the sole use of chest X-ray yielded a low sensitivity (64.2%) and a moderate specificity (73.6%) in detecting ILD. Similar results were reported by Hax et al. and applying a simple clinical decision rule developed by Steele et al. resulted in a sensitivity and specificity of 58.6 to 88.7% and 60.0%, respectively, in identifying ILD using physical examination or/and chest X-ray [34, 35].

### HRCT

In our study, the most common pulmonary HRCT findings in patients with IRD-ILD were GGO (36.5%) and NSIP (31.8%), followed by UIP (9.5%), as also described by Capobianco et al. [36]. According to Goldin, among other changes, pure GGO (pGGO) is also a common finding in SSc-ILD [37]. Given that these are initial diagnosis of IRD in our study, predominant GGO often present without fibrotic patterns (reticulations or honeycombs) as a correlate of beginning ILD. In our study, HRCT showed the highest sensitivity (100.0%) with a specificity of 55.3%. Thus, our results are consistent with the majority of studies, regarding HRCT generally as the gold standard for the diagnosis of ILD [8, 30, 31, 34, 35, 38, 39]. In addition, the evidence-based European consensus statements for the identification and management of ILD in SSc recommend that SSc patients should be screened for ILD using HRCT, particularly if they are showing one or more risk factors [20]. However, it should be emphasized that HRCT is highly sensitive in detecting pulmonary morphologic changes, but IRD patients do not necessarily have ILD despite the presence of these changes. That is the reason why patients were partially excluded in some studies [30, 31].

### Combination of pulmonary function test and imaging

The results of our study are in accordance with the literature, showing that a combination of several PFT parameters did not increase specificity without a significant loss of sensitivity in detecting ILD [30, 31].

We revealed a sensitivity and a specificity of 95.2% and 38.7% (positive LR 1.55 and negative LR 0.12), respectively, by using a combination of PFT (DLCO < 80%) and chest X-ray. Thus, a negative test result is 8.3 times more likely in non-ILD patients than in ILD patients. Also, in sub-analysis, we revealed a sensitivity and a specificity of 89.5% and 26.3% (positive LR 1.21 and negative LR 0.40), respectively, by using the same combination. These differences are caused by the facts that the sub-analysis is an already preselected group of patients, because the study was performed in Germany and, according to the recommendations, at least one risk factor should be present for performing a pulmonary HRCT [20]. Considering that, the loss of specificity of the DLCO can be explained. In summary, the sub-analysis shows comparable results. Also, in the representative clinical collective, similar results could be demonstrated, considering that there was no homogeneity in this collective.

Furthermore, the combination of TLC, TLCO, FVC, FEV_1_, and chest X-ray was associated with a lower sensitivity (64.5–80.6%), even in the sub-analysis. Steele et al. used chest X-ray or PFT (with FVC < 80% and FEV_1_/FVC > 70%). They could achieve a sensitivity and specificity of 60.5% and 77.3% with positive LR 2.67 and negative LR of 0.51, respectively [35]. Bernstein et al. and Suliman an co-workers showed a sensitivity and a specificity of 59.0 to 74.1% and 45.7 to 65.8%, respectively, with positive LR of 1.47 to 1.7 and negative LR of 0.36 to 0.6, by using a combination of FVC (< 80%) and DLCO (< 70% or < 80%) [21, 30]. With these algorithms, 25 to 40% of patients with ILD would be scored as false negative.

Furthermore, our study revealed a better sensitivity (83.6%) and specificity (83.1%) with the combination of PFT (DLCO < 80%) and HRCT. The best combination of sensitivity (95.2%) and specificity (77.4%) was observed for DLCO < 80% and/or suspicious chest X-ray findings, followed by HRCT (positive LR 4.21 and negative LR 0.06). Combining these examinations, a low rate of false-negative results could be achieved due to the high sensitivity. This procedure potential reflects a screening algorithm for the detection of IRD-ILD. Additionally, a screening algorithm in patients with newly diagnosed IRD should be highly sensitive with also low negative LR (even accepting a poorer specificity), because it concerns an already a pre-selected population with a high risk of pulmonary involvement and a high mortality over time.

A potential limitation of our study is the fact that we performed HRCT in IRD patients with a DCLO < 80%. Regarding the rules for the application of ionizing radiation, patients with a DLCO > 80% underwent no pulmonary HRCT or only in justified individual cases (other risk factors). Consequently, the diagnostic value of the presented algorithm could be potentially overestimated, because we did not perform HCRT on every study participant.

## Conclusions

ILD in patients with IRD is associated with increased morbidity and mortality, and a new effective treatment option is now available. Therefore, screening for lung involvement at the onset of IRD is crucial. By using a stepwise approach, we found DLCO combined with chest X-ray proved to be a potential screening tool for detecting early lung manifestations in IRD patients. Based on the high sensitivity of DLCO in combination with chest X-ray, all patients with a reduced DLCO (< 80%) or/and suspicious chest X-ray findings should undergo pulmonary HRCT to detect inflammatory activity in the lungs and to exclude other pulmonary differential diagnoses. Further studies should be initiated to verify these initial findings.

## Data Availability

The datasets used and/or analyzed during the current study are available from the corresponding author on reasonable request.

## Abbreviations

COP: Cryptogenic organizing pneumonia
COPD: Chronic obstructive pulmonary disease
CTD: Connective tissue disease
DLCO: Diffusing capacity for carbon monoxide
EGPA: Eosinophilic granulomatosis with polyangiitis
FEV_1_: Forced expiratory volume in 1 s
FVC: Forced vital capacity
GGO: Ground-glass opacities
pGGO: Pure ground-glass opacities
GPA: Granulomatosis with polyangiitis
HRCT: High-resolution computed tomography
ILD: Interstitial lung disease
IPF: Idiopathic pulmonary fibrosis
IRD: Inflammatory rheumatic diseases
LIP: Lymphoid interstitial pneumonia
MPA: Microscopic polyangiitis
NSIP: Non-specific interstitial pneumonia
PFT: Pulmonary function tests
PY: Pack years
RB-ILD: Respiratory bronchiolitis interstitial lung disease
ROC: Receiver operating characteristic
SLE: Systemic lupus erythematosus
TLC: Total lung capacity
TLCO: Transfer factor of the lung for carbon monoxide
UIP: Usual interstitial pneumonia
